# Thermal Stability, Flammability and Mechanical Performances of Unsaturated Polyester–Melamine Resin Blends and of Glass Fibre-Reinforced Composites Based on Them

**DOI:** 10.3390/polym14224885

**Published:** 2022-11-12

**Authors:** Latha Krishnan, Baljinder K. Kandola, Dario Deli, John R. Ebdon

**Affiliations:** Institute for Materials Research and Innovation, University of Bolton, Deane Road, Bolton BL3 5AB, UK

**Keywords:** unsaturated polyester, melamine formaldehyde, blend, cast resins, composites, thermal stability, fire properties, cone calorimetry, mechanical properties

## Abstract

A novel blend of unsaturated polyester (UP) resin with an inherently flame-retardant and char-forming melamine formaldehyde (MF) resin has been prepared with the aim of reducing the flammability of the former. MF resin, sourced as a spray-dried resin, was dissolved in diethyleneglycol solvent; the dissolved resin and the UP-MF blend were autocured by heating under conditions normally used for curing UP, i.e., room temperature for 24 h and post-curing at 80 °C for 12–24 h. The cured UP-MF blends, although heterogeneous in nature, were rigid materials having fire performances superior to those of the cured UP alone. The blends also burned, but with a much reduced smoke output compared with that from UP. Although the heterogeneity of the blends helped in improving the fire performances of the blends in terms of the MF domains forming a semi-protective char, acting as thermal barriers for the adjoining UP domains, and hence reducing their thermal degradation, the mechanical properties of composites based on them were impaired. Nevertheless, whilst UP/MF blends may not be suitable for use as matrices in glass-reinforced composites in load-bearing applications, they may lend themselves to applications as fire-retardant gel coats, especially in view of their low-smoke, char-forming attributes.

## 1. Introduction

Amongst thermoset resins used for fibre-reinforced composites, unsaturated polyester (UP) and epoxy resins are the most popular choices, mainly because of their good mechanical properties, and chemical and corrosion resistance [1,2]. UP has a further advantage of good moisture resistance and, hence, is popular in marine applications. UP, however, is highly flammable and burns with heavy smoke and soot, mainly due to its styrene component being released during thermally induced depolymerization reactions [1,2,3,4]. Commonly used halogen- and phosphorus-containing flame retardants, while reducing the flammability, have a minimal effect on smoke and soot production [1,2]. Inorganic additives such as alumina trihydrate, typically used at the >50 wt% level while reducing flammability and smoke production, cause processing problems and impair the mechanical properties of the derived composite [3,5]. An alternative approach is to blend the resin with an inherently flame-retardant and no/low-smoke-producing resin, such as a phenol formaldehyde or melamine formaldehyde resin [6].

Generally, formaldehyde-based resins are thermosetting adhesives, widely used in furniture, flooring, exterior cladding, and wood-based panel and laminate materials [7,8,9]. Melamine (1, 3, 5-triazine-2, 4, 6-triamine) formaldehyde (MF), owing to its beneficial properties such as high crosslinking density, cost-effectiveness, rapid curing, and self-extinguishing performance with minimal smoke emission and negligible toxicity, offers the additional benefit of being able to be used for flame-retardant applications [9,10,11,12]. Whilst phenol formaldehyde, simply called phenolic (PH) resins, are quite often used in glass/carbon fibre-reinforced composite applications where fire retardancy is an important criterion, MF resins are limited to wood composites [9,13].

Whilst resin blending works very well with chemically compatible resins, such as epoxy and phenolics [14], with incompatible resins phase separation may occur. However, there is the possibility that in thermoset resins physical mixing by mechanical stirring with a high shear force can lead to the formation of interpenetrating networks (IPNs), i.e., two or more networks that are at least partially interlaced on a molecular scale but that are not chemically bonded to each other [15,16]. It is important though that the resins are co-cured into the matrix for there to be good/acceptable physical and mechanical properties of the latter. In the case of UP and MF/PH systems, there is physical incompatibility in respect of the hydrophobicity of UP and hydrophilicity of the formaldehyde-based resins, coupled with incompatibility in their curing chemistries, i.e., UP resins cure through a free-radical chain reaction mechanism whereas MF/PH resins cure via polycondensation [17,18] and for MF [12] with the elimination of water. Despite these difficulties, we have shown previously that UP resins may successfully be blended and co-cured with phenolic resoles [17,18,19] and novolacs [20,21]. These co-cured resin blends were significantly more flame-retardant than unmodified UPs but had slightly impaired but still acceptable physical and mechanical properties for use in glass-reinforced composite laminates [22]. However, the curing process was multi-step, was long, required high-temperature curing, and was therefore not a commercially attractive solution. MF resins on the other hand are low-temperature curing resins; hence, they were chosen for this work.

Blending of MF with UP is more challenging than that of PHs, given that MF is water-soluble. Blending of MF with polymers such as polyvinyl alcohol (PVA) and polyethylene glycol (PEG) has previously been reported [11,23], where it was shown that low compatibility between MF resin and PVA at room temperature results in phase separation, thereby impairing the mechanical properties of the composite material. To overcome this, compatibilizers have been used [11,23]. Blending of MF with UP, though, has never been attempted. In the case of UP-PH blends, we have reported compatibilization of resoles using different approaches: (i) the use of a common solvent (ethanol), (ii) using an external compatibilizer (epoxy functionalised phenolic resin), and (iii) chemical functionalization of at least one of the components of the blend (use of an allyl functionalized phenolic resin) [17,18,19]. Of the modified resoles, that bearing allyl groups (a commercial Methylon resin) was found to be the most satisfactory in forming homogeneous blends with UP owing to the chemical incorporation of at least some of the allyl groups into the free-radically crosslinked network structure, resulting in very good physical and mechanical properties. However, the least compatible resin blend (UP and unmodified PH resole) showed better flame retardance than the most compatible UP and allyl-PH resole blend [18]. We believe that the reason for this is that in the least compatible blend, the cured PH domains are uniformly dispersed within a matrix of cured UP, the former acting as nucleating centres for char formation in the surrounding UP matrix, thus providing a greater thermally protective effect for the latter [18]. Because of these findings, use of an unmodified MF rather than any chemically modified MF for reducing flammability of UP is explored here.

## 2. Materials and Methods

### 2.1. Materials

Unsaturated polyester (UP) resin: Crystic 2-406PA (Scott Bader, Wellingborough, UK), a pre-accelerated, thixotropic condensate of phthalic anhydride, propan-1,2-diol, and maleic anhydride containing 35–40 wt% styrene and 0.2 wt% cobalt octoate accelerator.

Free radical initiator for UP curing: Catalyst M (Scott-Bader, Wellingborough, UK): a *ca* 50 wt% solution of methyl ethyl ketone (MEK) peroxide in MEK.

Melamine formaldehyde resin: A proprietary spray-dried melamine formaldehyde resin, SD-MF, was received from a commercial source. The resin was dissolved in diethyleneglycol solvent (Sigma Aldrich) prior to its use, identified as MF resin in this work.

Glass fibre: Woven roving E-glass fibre, 300 g/m^2^ (Glasplies, Southport, UK).

### 2.2. Casting and Curing of Resin Plaques 

A sample of cured UP resin was prepared by mixing the resin with 2 wt% of catalyst M by continuous hand stirring for around 5 min in a 100 mL beaker. The mixture was then poured into round moulds (5 cm diameter, 3 mm depth). The resin was then cured at room temperature for 24 h and post-cured at 80 °C in an oven for 6 h (Table 1). These curing conditions used for UP are similar to those reported in our previous publications [18,20].

MF cast resins were prepared by dissolving the spray-dried melamine formaldehyde resin powder (SD-MF) in diethylene glycol solvent (DEG) (1:1 weight ratio). The mixture of SD-MF and DEG is considered a pure melamine formaldehyde resin (MF) in the following discussions. The MF resin was directly transferred to 55 mm diameter moulds to depths of 3 mm and cured using conditions given in Table 1. A slow heating rate of 2 °C/min was used in the oven to cure the resin with different curing cycles because a fast heating rate will cause the sudden release of water/formaldehyde during the curing process causing bubbles and voids. The curing/post-curing conditions of both MF resins and their blends with UP discussed below were established by differential scanning calorimetry (DSC), discussed in a later section.

Resin blends were prepared by mixing 70:30 and 50:50% *w*/*w* UP/MF for 10 min in a 100 mL beaker using a high-speed, overhead, electric stirrer fitted with a four-component blade (IKA RW16 (Merck Life Cycle UK Ltd., Dorset, UK) at 900 rpm). In order to produce samples without bubbles, which could be caused by the trapped air, the resin was degassed under vacuum for 10 min. Catalyst M (2% by weight with respect to resin blend) was then added to the resin mixtures and stirring continued for a further 10 min. The resulting mixtures were transferred to moulds and cured using conditions given in Table 1. 

### 2.3. Composite Preparation

Glass fibre-reinforced composites (GFRC) of pure UP, MF and UP/MF:50/50 were prepared using the hand lay-up and vacuum bagging technique. Eight layers of woven E-glass fabric (300 mm × 300 mm), impregnated individually with resin (50% each by weight), were stacked, vacuum bagged, and cured using curing conditions given in Table 1. The mass fraction of resin and the glass fibre was similar in all three composites (55–58% glass fibre and 42–45% resin), the laminate thickness was ~2.5 mm. All three composites were visually good with uniform plain surfaces without any voids.

### 2.4. Characterisation of Resins

Attenuated total reflectance Fourier transform infrared (ATR-FTIR) spectroscopy was performed on cured samples using a Nicolet iS10 spectrometer equipped with a Smart iTR attachment employing a single bounce diamond crystal.

Differential scanning calorimetry (DSC) was used to monitor the curing of resin samples (2–10 mg) at a heating rate of 5 °C min^−1^ over the temperature range 30–350 °C using a TA Instruments Q2000 (Waters, New Castle, UK) differential scanning calorimeter.

Dynamic mechanical thermal analysis (DMTA) was carried out on a TA Instruments Q800 DMA (Waters, Newcastle, UK) machine using a single cantilever clamp and multi-frequency-strain set-up (0.1% strain and 1 Hz frequency). The specimens were heated at 10 °C min^−1^ within the temperature range 30–350 °C. Values of tan δ and storage modulus were recorded.

Scanning electron microscopy (SEM) was performed on small samples of cured, cast resins previously fractured in simple bending experiments, and the fracture surfaces then gold coated using a Polaron Range SC7620 Sputter Coater with 60 s plasma exposure. The coated fracture surfaces were examined using an Hitachi S-3400N (Manchester, UK) variable pressure scanning electron microscope.

Thermal stabilities of cured resins and their blends were assessed by thermogravimetric analysis (TGA) using a TA Instruments SDT 2960 (Waters, Newcastle, UK) over the temperature range 25–800 °C using 15 ± 1 mg samples heated at a constant rate of 10 °C /min in both air and nitrogen flowing at 100 ± 5 mL/min. In order to demonstrate the effect of the charring MF resin on residual char in the UP/MF blends with a view to reduce the flammability of the UP, the char yields of all samples at 575 °C have been compared. This temperature was selected as UP resin decomposes completely beyond 575 °C, leaving no char residue in air. 

In addition to the analyses of TGA curves of UP, MF, and their blends, theoretical degradation curves for both blends were obtained using the rule of mixtures and compared with experimental curves in order to highlight any interaction between the two components. The theoretical curves were obtained using the formula: calculated mass of the blend at each temperature point = (mass fraction of UP × measured mass of cured UP) + (mass fraction of MF × measured mass of cured MF). This method has been previously reported to observe such interactions between different components in a formulation [24,25].

During the experiments in nitrogen, the SDT 2960 was linked to a Nicolet Smart iS10-iTR FTIR spectrometer (ThermoFisher Scientific, Waltham, MA, USA) equipped with a gas cell for the analysis of gases evolved during decomposition

### 2.5. Flammability Assessment of Cast Resins and GFRCs

Limiting oxygen indices (LOI) of cured resins were measured by a standard method (BS 2782) using a Fire Testing Technology (FTT, East Grinstead, UK) LOI instrument equipped with an oxygen analyzer.

The flammability of cast resin samples and GFRC composite laminates samples were evaluated by a cone calorimeter (Fire Testing Technology, East Grinstead, UK) in accordance with ISO 5660. The circular cast resin samples of 55 mm diameter with a 3 mm thickness were used, whereas for composite laminates 75 mm × 75 mm specimens with a thickness of approximately 2 to 3 mm were used. Before testing, the bottom surface and the edges of the samples were wrapped with aluminium foil to ensure that only the top surface would be directly exposed to the heat source. A minimum of three tests were performed for each formulation by exposing them to 50 kW/m^2^ incident heat flux in the horizontal mode with an ignition source.

### 2.6. Mechanical Property Measurements of GFRCs

A three-point bending flexural test was carried out according to BS EN ISO 14125 using an Instron 3369 universal testing machine (Instron, High Wycombe, UK). A 100 N load cell with a compression rate of 1 mm/min was used on the samples with a span length of 100 mm. Tests were undertaken within the elastic range of the material due to limited number of samples. Three replicate specimens of the size 150 mm × 20 mm × ~2.5 mm thickness were tested, and the results were averaged.

Tensile testing was carried out according to BS EN ISO 527 using an Instron 3369 (Instron, High Wycombe, UK) universal testing machine. The gauge length of each specimen was 100 mm, and polymeric tabs were bonded at their ends to improve the gripping and to ensure failure within the gauge region. The tests were conducted using a 50 kN load cell with a crosshead speed 1 mm/min. Tensile modulus and strength values were calculated form stress–strain curves using an extensometer, and selective samples had strain gauges bonded to their surfaces to verify the results. Three replicate specimens of the size 150 mm × 20 mm × ~2.5 mm thickness were tested, and results were averaged.

The impact properties of the samples were investigated using an Instron Dynatub Mini-Tower drop weight impact machine (Instron High Wycombe, UK) in accordance with ASTM D7136 The samples, sized 75 mm × 75 mm, were fully clamped circumferentially by a 50 mm diameter holder. The clamped samples were impacted by dropping a steel 16 mm diameter hemispherical impactor from 100 mm height to create an impact energy level low enough to avoid significant surface damage so further testing of the samples could be carried out for post-fire impact testing. A high-speed data acquisition system (Dynatup^®^ Impulse^TM^ software data capture system) was used to obtain load–central displacement curves. Three replicate specimens of each sample were tested, and the results were averaged.

## 3. Results and Discussion

### 3.1. Curing Behaviour Study by DSC 

**Figure 1:**
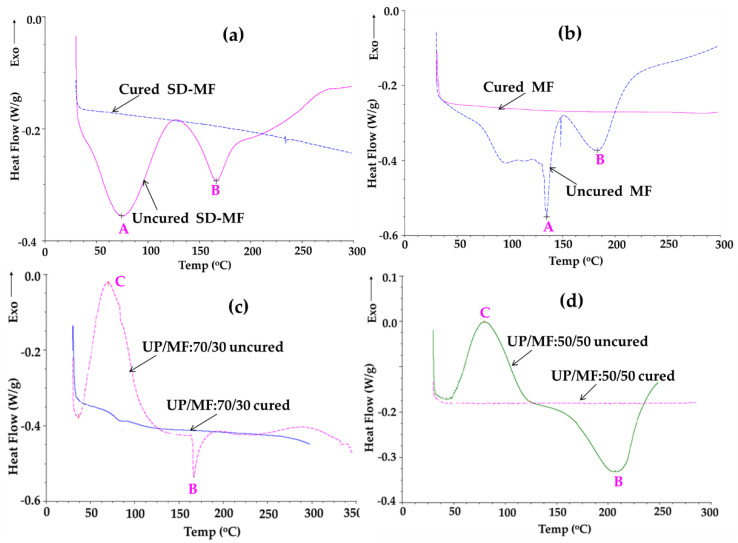
DSC curves of uncured and cured (**a**) SD-MF powder, (**b**) MF, (**c**) UP/MF:70/30, and (**d**) UP/MF:50/50.

Since in this work, spray-dried melamine formaldehyde (SD-MF) resin was dissolved in DEG and the effect of DEG on curing behaviour is not known, the curing behaviour of SD-MF in the absence of any solvent was also studied by DSC. Figure 1a shows the DSC curves of uncured and the cured SD-MF resin, with the latter having been subjected to a previous heating cycle in the DSC at 30–300 °C to bring about its curing. The uncured SD-MF shows two endothermic peaks with T_peak_ at 74 °C and 166 °C, indicating that SD-MF has two stages of curing [12]. The detailed analyses of the peaks are given in Table 2. The first endothermic curve may be attributed to the formation of methylene ether links from pairs of methylol groups with the release of water and the second endothermic curve is attributed to the cross linking reaction (condensation reaction with the elimination of formaldehyde/water) [12]. The evolution of water causes a strong endothermic signal which masks the exothermic cross-linking reaction (see Figure 1a) [26]. In the DSC scan of the previously heated sample, the disappearance of curing peaks indicates that the resin is completely cured.

The DSC curve of uncured MF resin (SD-MF dissolved in DEG) also shows two endothermic peaks (Figure 1b), but the T_peaks_ have shifted to a higher temperature compared to those in SD-MF in the absence of DEG, indicating the effect of DEG, which has a boiling point of 245 °C and, hence, will still be present in the resin up to that temperature. 

**Scheme 1:**
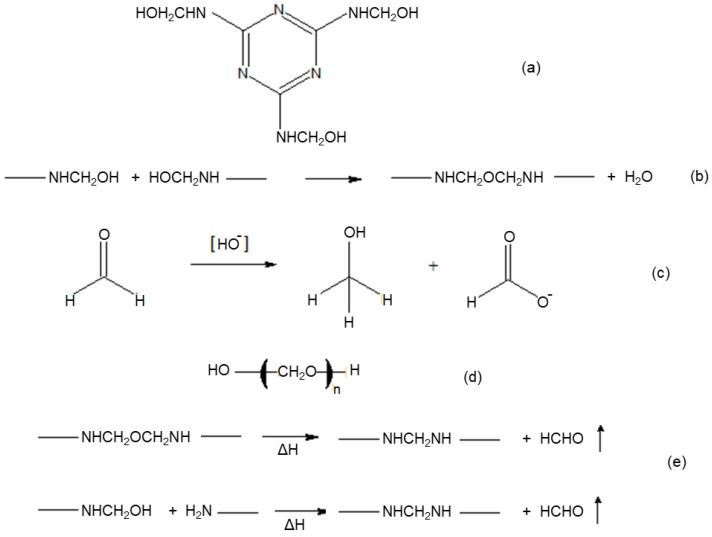
(**a**) Trimethylolmelamine, (**b**) formation of methylene ether linked oligomers, (**c**) Cannizzaro reaction, (**d**) formation of formaldehyde oligomers, and (**e**) formation of methylene links.

SD-MF is a standard (non-methylated) melamine–formaldehyde resin made by condensation under alkaline conditions of melamine with aqueous formaldehyde in a molar ratio of 1:3. As first prepared, it is a 50% solution in water of a mixture of low molecular weight methylolmelamines in which N, N’, N”-trimethylolmelamine (Scheme 1a) can be expected to be the principal component, given that primary amine (-NH_2_) groups react more readily with formaldehyde than secondary (-NH-) ones [27]. However, a variety of methylolmelamines can be expected to be present ranging from monomethylolmelamine, N,N- and N,N’-dimethylolmelamines through to penta and hexamethylolmelamines [28]. There will probably have been also a little condensation of methylolmelamines to give methylene ether linked dimeric, trimeric, tetrameric species, etc. [28]. Following preparation, SD-MF has been spray-dried to give a powder, during which further condensation of methylolmelamines will undoubtedly have occurred to give higher methylene ether linked oligomers (Scheme 1b). After this drying stage, the overall average molecular weight is expected to be no higher than 2000.

No acid catalyst or other additive has been added to the SD-MF. However, when SD-MF is heated, it can be expected to autocure because any free formaldehyde in the resin is likely to be converted to formic acid (which acts as an acid polymerisation catalyst) in a Cannizzaro reaction (Scheme 1c). There is another possibility: free formaldehyde will be present in its monomeric form and can polymerise to give oligomers such as paraformaldehyde (Scheme 1d). Chain extension and crosslinking in melamine–formaldehyde resins occurs initially via the formation of methylene ether links from pairs of methylol groups with the release of water (Scheme 1b), but at longer reaction times and higher temperatures, methylene links may be formed from methylene ether links with the release of some formaldehyde or by further reactions of methylol groups with amine groups (Scheme 1e). 

**Scheme 2:**
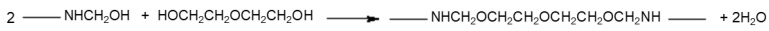
Reaction between SD-MF and DEG.

SD-MF can also be expected to react to an extent when heated with DEG. Again, this is an acid-catalysed reaction and will proceed via the same mechanism as in the reaction of methylolmelamines with methanol to give methylated resins. Since DEG is a difunctional alcohol, it can act as a crosslinking agent in reactions with methylolmelamines (Scheme 2). When mixtures of SD-MF with DEG are heated in an open system, the release of water, formaldehyde, and any unreacted DEG can occur.

The curing behaviour of UP has been studied in details in our previous communications [17,18,19], showing a pronounced exothermic peak between ca. 30 and 140 °C with a maximum at about 82 °C (Table 2), representing curing reaction.

The DSC curve of uncured UP/MF:70/30 indicates a two-step curing reaction, the first exothermic with a T_peak_ of 74 °C, with a heat of reaction of 196.9 J/g, both of which resemble the DSC exothermic curing peak of UP (T_peak_ 82 °C with a heat of reaction of 282 J/g). The first stage of the endothermic reaction in MF has been masked by the exothermic reaction of UP because of its 70 wt% presence in this blend. The second stage of MF curing gives an endothermic curve, with a T_peak_ of 167 °C (see Table 2).

The second stage of MF curing is a cross linking condensation process with the evolution of water/formaldehyde at temperatures between 137–200 °C [28,29,30]. The UP/MF:70/30 blended resin is cured first at room temperature for 24 h and then at 80 °C for 24 h. After curing, the DSC trace of the UP/MF:70/30 resin is a flat line indicating that the resin is 100% cured (see Figure 1c). In the case of the UP/MF 50:50 blend, the first exothermic peak is similar to that of the 70:30 blend, although the heat of reaction is considerably reduced (88 J/g in the 50:50 blend compared to 197 J/g in the 70:30 blend). The second endothermic reaction, indicating the cross linking stage of MF resin with a T_peak_ of 207 °C, is at a higher temperature than in UP/MF:70/30 (166 °C) and is also broader (because of the higher MF content as well as the presence of DEG).

These DSC results were taken as the guide points in establishing the optimal curing conditions for the resin samples presented in Table 1. DSC traces of all the cured samples in Figure 1b–d are flat lines, indicating that the resins are completely cured.

### 3.2. Characterisation of Cured Resins and Resin Blends 

#### 3.2.1. Chemical Characterisation by Infrared Spectroscopy

**Figure 2:**
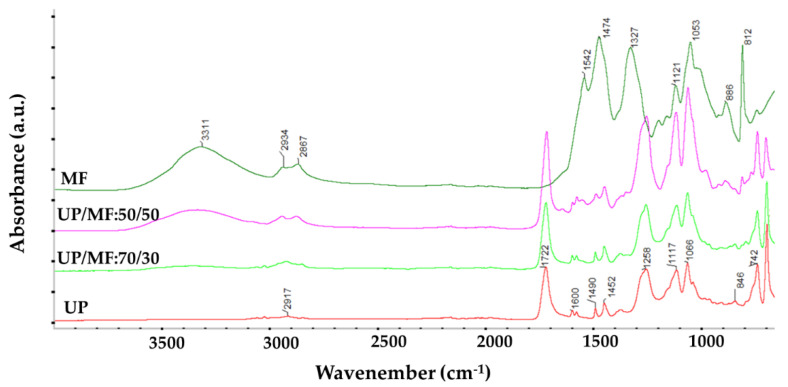
IR-ATR spectra of UP, UP/MF:70/30, UP/MF:50/50, and MF cured cast resins.

IR-ATR spectra of UP, MF, UP/MF:70/30, and UP/MF:50/50 cured resin samples are shown in Figure 2. The spectrum of UP displays bands at 2985 cm^−1^ for unsaturated =C-H stretching vibrations and 2917 cm^−1^ for aliphatic hydrocarbon stretch, but the most characteristic band of UP is the strong carbonyl stretch at 1722 cm^−1^. Bands at 1600 cm^−1^ and 1490 cm^−1^ arise from aromatic rings. In MF, the band at 3311 cm^−1^ is assigned to the NH stretch of primary amine attached to the methylene bridge [31,32]. The peaks at 2851 and 2924 cm^−1^ are attributed to the methylene C-H symmetric and asymmetric stretch. The peak at 1542 cm^−1^ can be assigned to N-H bend of bridging secondary amine [31,32]. The peak at 1474 cm^−1^ can be attributed to the methylene C-H bend. The peak at 1121 cm^−1^ may be assigned to secondary amine [31]. The peak at 813 cm^−1^ is the characteristic signature of a triazinyl ring [32].

The IR spectrum of UP/MF:50/50 contains all the peaks characteristic of UP as well as of MF. In UP/MF blends, the 3311 cm^−1^ peak area is broader and the intensity of the bands are reduced compared to those in pure resins as expected, but it is difficult to quantify the content of resin in the blends using the intensity of the bands or to identify any reaction between the components of the blended resin.

#### 3.2.2. Compatibility Study by DMTA

**Figure 3:**
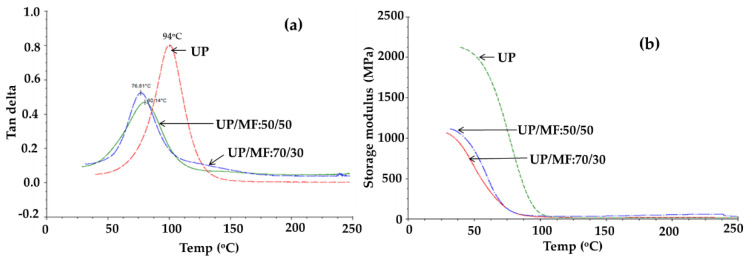
(**a**) Tan δ and (**b**) storage modulus vs. temperature curves for cured resin of UP, UP/MF:70/30, and UP/MF:50/50.

Plots of tan δ vs. temperature for UP, UP/MF:70/30, and 50/50 blended cured cast resins are given in Figure 3a; from the peak temperatures, their *T_g_* values have been identified and are reported in Table 3. It was not possible to carry out DMTA for MF because it was too brittle. The UP/MF:70/30 and 50/50 blends show single tan δ peaks similar to that of UP, indicating that the blended materials behave as single homogeneous materials or that the *T_g_* of the pure UP and MF are similar. The *T_g_* of MF has been reported to be around 70 °C by Cai et al. [33]. The *T*_g_ of UP is 94 °C, which is reduced to 77 and 80 °C, respectively, in the blends. 

**Figure 4:**
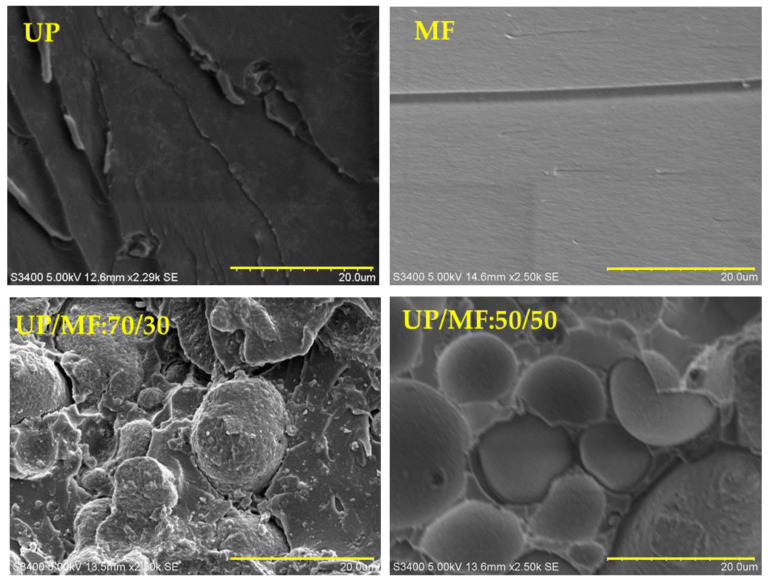
SEM images of fractured surfaces of UP, MF, UP/MF:70/30, and UP/MF:50/50 cured resin.

As seen from Figure 3b, the storage moduli of all the samples decrease on increasing the temperature from 40 °C to around 110 °C. The plateau above 110 °C indicates that all the samples were completely cured. The storage moduli of the blends are lower than that of UP, as seen from Table 3, with the value of UP/MF:70/30 being lower than that for UP/MF:50/50. The difference between the values of the UP/MF blends could be because of the samples being cured to slightly different extents. However, the important observation to note is that the incorporation of MF lowers the modulus of UP. This indicates that MF may be acting as a plasticising agent, affecting the cross linking density of the overall resin, which decreases as the content of the UP is decreased. The poor storage moduli of the blends are due to the formation of a looser; are due to less crosslinked networks between the UP and the MF (because of a plasticising effect of MF); or alternatively, might be a consequence of micro phase separation between the components of the blended material, as apparent in the SEM images of fractured surfaces of cast resins shown in Figure 4.

Pure UP and MF exhibit apparently homogeneous, smooth, plain surfaces in Figure 4. However, heterogeneous morphology is noticeable in the images of the fracture surfaces of UP/MF:70/30 and 50/50 samples. It can be seen in Figure 4, that nearly spherical shaped MF domains are embedded in irregularly shaped UP domains in the blended resins. When the content of MF increases, the number of spherical shaped domains increases. It is possible that UP and MF resins in the blends cured simultaneously but as two different phases.

### 3.3. Thermal Stability of Resins and Resin Blends

Thermal stabilities of UP, MF, and their blended resins were investigated by thermal analysis. The thermogravimetric (TGA, mass loss versus temperature), DTG (derivative of TGA curves), and differential thermal analytical (DTA, temperature difference between reference and sample versus temperature) curves for all sample are given in Figure S1, and the data derived from these curves are presented in Table 4.

The TGA analysis of UP has previously been discussed in detail [18,20]. In summary, in both air and nitrogen up to ca. 180 °C, there is 0.9% mass loss, which can be attributed to volatilization of absorbed moisture, solvent and/or any unreacted monomers. In nitrogen, there is a single-stage mass loss between 183 and 462 °C with 94.8% mass loss, representing decomposition of the resin in which polystyrene cross-links decompose releasing styrene and other volatiles, and the residual polyester backbone degrades [18,20,34]. This single-stage decomposition is also corroborated by a single endothermic DTA peak at 369 °C (Table 4). In air, the decomposition stage is similar, but there is an additional stage in the temperature range 435–566 °C (with DTG maximum at 532 °C) with 5.6% mass loss, representing solid-state oxidation of char [18,20,34]. The decomposition stage in air is corroborated by a small endothermic DTA peak at 352 °C, although this is overlapped by a subsequent large exothermic DTA peak with a maximum at 404 °C, representing oxidation of volatile degradation products. The char oxidation stage is represented by an exothermic peak with a maximum at 533 °C. The resin decomposes completely beyond 575 °C, leaving no char residue in air. The detailed mechanisms of these reactions have been discussed elsewhere [18].

TGA of MF in air shows four stages of mass loss. The first stage involves 6.3% mass loss probably mainly due to evaporation of solvent/moisture and loss of volatiles during the post-curing from RT to 110 °C. The major polymer mass loss (39.4%) occurs in the second stage above 110 °C and up to 288 °C. This mass loss is due to the degradation of polymer as well as the additional DEG solvent evaporation (because MF is a mixture of SD-MF and DEG in 1:1 ratio and the boiling temperature of DEG is 245 °C). This second stage can be corroborated by the DTG peak at 218 °C and a small endothermic DTA peak at 216 °C. Further polymer degradation occurs in a third stage over a narrow temperature range of 288–382 °C with a mass loss of 25.7%, a DTG peak at 356 °C and an endothermic DTA peak at 378 °C. The last stage of mass loss representing char oxidation takes place over a wider range of temperature of 382–639 °C with a mass loss of 26%, a DTG peak at 597 °C, and an exothermic peak with a maximum at 593 °C (Table 4). The residue at 575 °C for MF is 13.1 wt% compared to 0.4% in UP. In nitrogen, there are also four stages of mass loss: In the first stage, there is 2.3% mass loss, which is less than in air. The mass loss in the second stage is also slightly less than in air (31.7% vs. 39.4% in air) but greater in the third stage (36% vs. 25.7% in air). The mass loss in the last stage (383–524 °C) is much reduced (9% vs. 15.6% in air), with a DTG maximum at 472 °C and an endothermic DTA peak at 474 °C as opposed to a large exothermic peak at 593 °C in air arising from oxidative degradation. The char residue at 575 °C in nitrogen is 21.5% which is more than that in air (13.1%) (Table 4), as expected.

The UP/MF:70/30 blend in air has three stages of mass loss similar to those in UP. The first stage is a small mass loss of 5.1% between room temperature and 206 °C, probably associated with dehydration or solvent evaporation. The second stage of significant mass loss occurs between 206 °C and 437 °C, with a mass loss of 78% and DTG max of 363 °C, the behaviour of which is close to that of UP. In UP/MF:70/30, the third stage of mass loss is only 15.6% between 437 °C and 605 °C and with a DTG maximum at 559 °C associated with the exothermic peak at 556 °C in the respective DTA curve (Table 4). However, the residue at 575 °C is only 4.4% which is higher than that of UP (0.4%). The mass loss rate in the thermo-oxidative degradation stage (third stage) is higher in blends, as shown by larger DTG peaks, indicating greater oxidation, similar to that in MF. The TGA curve of the 70/30 blend shows the overall improvement in thermal stability brought about by the MF compared with that of pure UP with the main improvement being in the greater char formation and in the higher temperature of third stage of degradation. The UP/MF:50/50 blend exhibits similar trends in its TGA, DTG and DTA curves (see Figure S1). As can be seen from Table 4, the residue at 575 °C increases with an increase in MF content of blends; the residue for UP/MF:50/50 being 7.4%. In nitrogen, the TGA and DTG curves of UP/MF:70/30, and 50/50 blends indicate two stages of mass loss, with the behaviour being between of those of UP and MF. In these blends, thermal stability is improved with the MF component acting as a char former. As in air, the blends have higher char yields than that of UP (see Table 4). Char yield increases with an increase in MF content.

**Figure 5:**
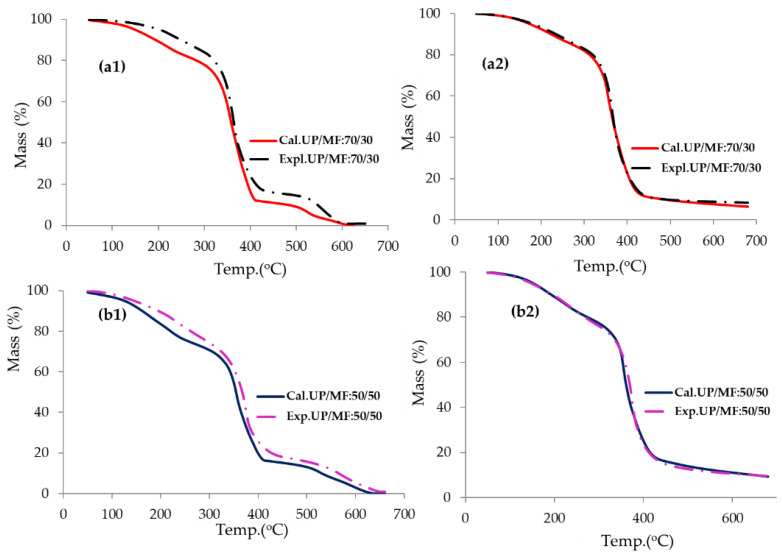
Experimental and calculated TGA curves for UP/MF:70/30 and UP/MF:50/50 cured resins in (**a1**,**b1**) air and (**a2**,**b2**) nitrogen.

In Figure 5 calculated mass losses ((mass fraction of UP × measured mass loss of cured UP) + (mass fraction of MF × measured mass loss of cured MF)) are given for UP/MF:70/30 and 50/50 blends in air and nitrogen in order to identify differences between these and the experimental results (actual mass loss from TGA test) and, thus, to highlight any synergistic or antagonistic interactions between UP and MF. It can be seen that there is a slight difference in the experimental and calculated curves in air for UP/MF:70/30 and 50/50 blends than in nitrogen. In air, the experimental mass loss is slightly less than the calculated values from room temperature to around 600 °C in both blends. In air, the blends of UP/MF:70/30 and 50/50 have shown slightly higher thermal stability than UP as expected. In nitrogen, on the other hand, there is no significant difference between experimental and calculated curves for both blends, indicating there is no reaction between the blend components. The DSC, DMTA, and SEM results indicate that the two resins are not intimately mixed; this study further shows that there is no chemical interaction between the decomposing components or their degradation products.

#### Evolved Gas Analysis

**Figure 6:**
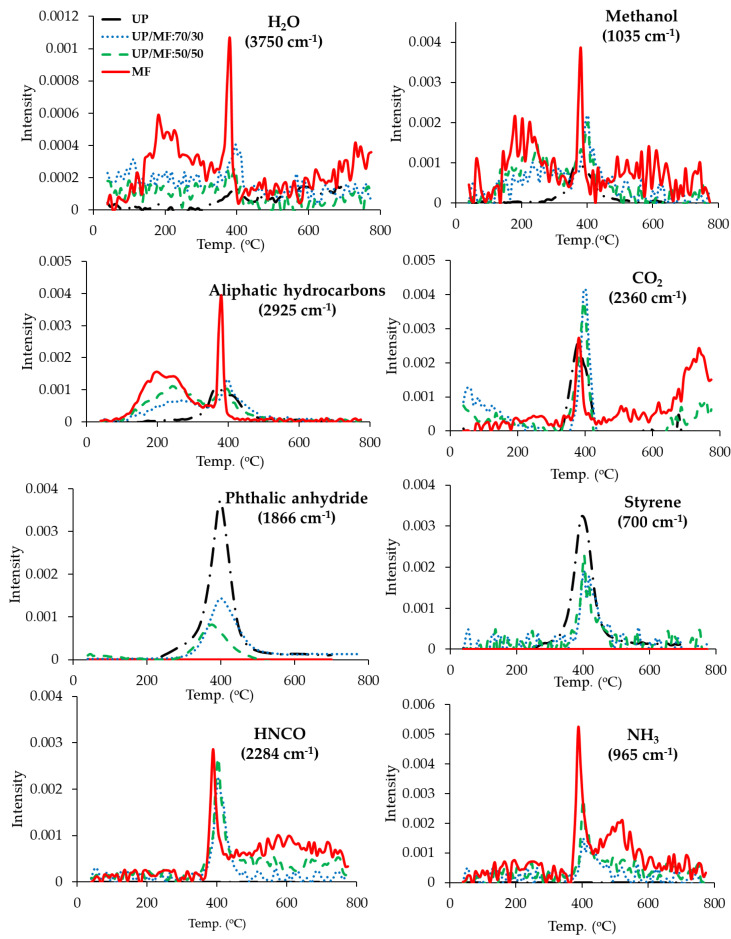
Absorbances of pyrolysis products for UP, MF, and UP/MF blends as a function of temperature obtained from TGA-FTIR in nitrogen.

The gases that evolved during the thermal decomposition of UP, MF, and their blends in nitrogen were analysed by TG-FTIR in an attempt to elucidate the thermal degradation mechanisms. Figure S2 shows the IR absorbance spectra recorded for volatile products of degradation of MF and UP/MF:50/50 blend at different temperatures in nitrogen. The intensities of bands in these spectra and those of other resins and resin blends were used to construct the plots of amount of degradation product versus temperature presented in Figure 6.

The main band assignments for UP have been discussed elsewhere [18,20] and are consistent with the evolution of carbon dioxide (2360 cm^−1^); phthalic anhydride (1866 cm^−1^); styrene (709 cm^−1^); and compounds containing aliphatic groups (2925 cm^−1^), benzenoid groups (1600 cm^−1^), and aromatic groups (3025 cm^−1^).

**Scheme 3:**
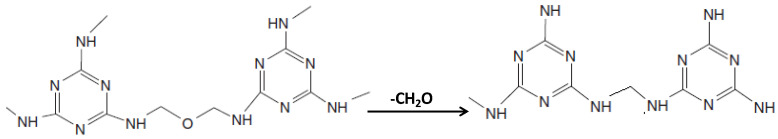
Formation of formaldehyde by the decomposition of methylene ether bridges [12].

For MF, the main evolved gases in the first and second mass-loss stages between RT and 287 °C are identified as water (3750 cm^−1^, O-H stretching vibration), methanol (1035 cm^−1^, C-O stretching vibration), CO_2_ (2360 cm^−1^), and compounds containing aliphatic groups (2925 cm^−1^, C-H stretching vibration). These evolve due to dehydration of absorbed moisture, post-curing, or initial degradation of MF, as discussed earlier in the TGA section. The amounts of gases evolved during the thermal degradation of the MF, UP resin, and their blends, calculated from the area under the curve of absorbance vs. temperature curves of each gas component, are listed in Table 5. It should be noted, however, that these amounts will be only approximate since the extinction coefficients for the various products are not known and may well be different. During the third stage of degradation between 287 °C and 383 °C, the main volatile products are HNCO, NH_3_, water, methanol, and a small quantity of formaldehyde. Formaldehyde is identified by a small peak within the band 1720 and 1740 cm^−1^ (see Figure S2b), and it is formed as shown in the Scheme 3. It was not possible to calculate the amount of formaldehyde because its peak was overlapped with the peaks from UP in the blends. From Figure S2b at 360 °C, it can be clearly seen that there is a broad peak between 2200 and 2400 cm^−1^ arising from the emission of HNCO gas, the characteristic band being at 2284 cm^−1^ [35]. The emission of NH_3_ is also confirmed in the fingerprint region by the H-N-H wagging band at 965 cm^−1^ and arises from the breakdown of the three-dimensional polymer structure [36]. During the fourth stage of degradation between 383 and 524 °C, the emission of HNCO and NH_3_ continues and ceases above 600 °C (see Figure 6).

In UP/MF resin blends, all the gases evolved during degradation of UP and MF resins in nitrogen are found also in the gaseous degradation products from UP/MF resin blends, as can be seen from Figure S2c and Figure 6. However, while the yields of most products from the blends, such as methanol, phthalic anhydride, styrene, CO_2_, HNCO, and NH_3_ lie between those of respective resin components (see Table 5), there was no new volatile species identified in the decomposition products of the blended materials, indicating that each component in the blends decomposes independently rather than there being an interactive decomposition of both resins. In Table 5, the calculated expected sums of the yields of volatiles of the UP/MF blends (calculated from the yields of individual components) are also given. As can be seen, there is no significant difference between calculated and measured yields, indicating that the resins exist as separate phases and are not intimately mixed.

### 3.4. Flammability of Cast Resins and Composites

**Figure 7:**
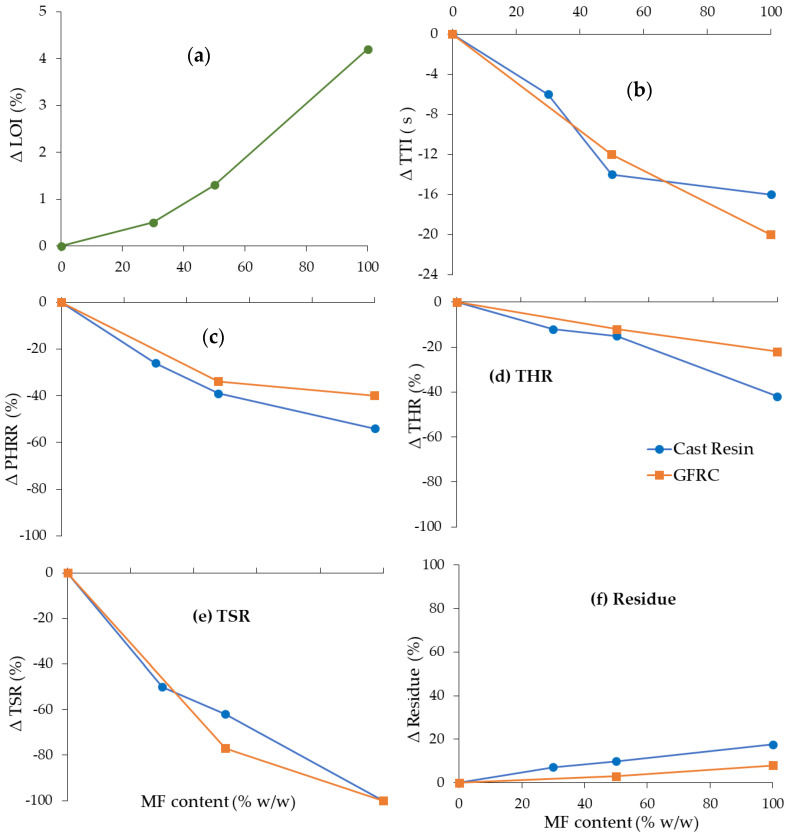
Effect of MF content on percent change in (**a**) LOI and (**b**–**f**) cone calorimetric parameters with respect to the respective values for UP in UP/MF cast resins and GFRC composites.

The flammability behaviours of UP and MF and the effect of MF on the flammabilities of the UP/MF blends in the form of cast resins were initially assessed by an LOI test. The LOI values of pure UP, MF, and UP/MF blends are listed in Table 6 along with calculated LOI values for the blends based on those of the components of the blends. The LOI of MF is 22.1% and is higher than that of UP (17.9%). The LOI values of UP/MF:70/30 and 50/50 blends (18.4 and 19.2%, respectively) lie between those of UP and MF, as also clearly seen from Figure 7a, increasing with increasing MF content. In order to investigate any interaction between the UP and MF in their blends, the LOI values were calculated with respect to the mass fraction of the components in the blends, assuming a rule of mixtures applies. It can be seen that the calculated LOI values are higher than the experimental ones, indicating that there is no interaction or reaction between UP and MF resin in the blends (a finding supported by the DMA and SEM results); rather, there is some antagonistic effect between the components. This pattern is commonly seen between incompatible resins, as noted previously for UP-phenolic resole blends [18] and reported also by others [37]. The unblended domains of UP may act as source of ignition, resulting in lower than expected LOI values. In homogenous blends, additive or synergistic behaviour is seen as previously reported for UP-methacrylated novolac blends [20].

The cured cast resin samples of UP, MF, and their blends were also subjected to cone calorimetric tests at 50 kW/m^2^ heat flux and their heat release rates (HRR), rates of smoke release (RSR), and percentage mass losses recorded. The HRR, % mass loss and RSR versus time curves for UP, MF, and their blends are shown in Figure S3, and the derived parameters are listed in Table 6. 

MF was ignited at 20 s, which is earlier than that of UP (36 s); this might be due to the higher solvent (DEG)/volatiles content of MF. The pure MF resin burns for a slightly longer time (198 s) than UP (188 s) but with a much lower PHRR (513 kW/m² vs. 1110 kW/m²) and with a much reduced total heat release (THR) (48 MJ/m^2^) than for the latter (83 MJ/m^2^). The slow combustion behaviour of MF is also evident from the shape of the HRR curve in Figure S3a1. PHRR and THR for MF are 54% and 42% lower than those of UP. The most striking feature is that there is no smoke production in MF. MF resin crosslinks at higher temperature, resulting in a highly crosslinked char residue that is rich in both carbon and nitrogen, which acts as a thermal barrier similar to its action in phenolic resins [37,38], and with evolution mainly of small hydrocarbon fragments that readily combust. As can be seen from Table 6, the char yield of MF is 18.9% compared to 1.3% in UP.

The UP/MF:70/30 and 50/50 blends show TTI of 30 s and 22 s, respectively, i.e., decreasing with increase in MF content. This is due to its high solvent/volatile content of MF. This can also be seen from Figure 7b, where ∆ TTI (TTI value of sample—TTI of UP) vs. MF content are plotted. These results support the lower than expected LOI values of the blends, as in the LOI test, the ignition behaviour is the determining factor. Flameout times of the blends also slightly increased owing to their MF content (see Table 6). From Figure S3, it can be seen that HRR, smoke release, and mass loss curves lie in between those of UP and MF. In order to better observe the effect of MF in the blends, percentage changes in PHRR, THR, TSR, and char residues with respect to the respective values for UP are plotted as a function of MF content in Figure 7b–f. As can be seen from Figure 7c, PHRR decreases with increasing MF content. THR is very similar for 70/30 and 50/50 blends (Table 6 and Figure 7d) and is only slightly lower than that of UP (12–15% reduction, Figure 7d). Similarly, there is not much effect on char residues either, as seen from Figure 7f. These results are similar to the cone calorimetric results for phenolic resins and UP/phenolic blends reported previously [18], although they are unexpected from inherently flame-retardant resins. It must be noted, however, that in cone calorimetric tests, there is forced combustion; hence, these resins do not behave in the same manner as in other flammability tests such as measurements of surface flame spread. The most interesting results are for smoke production in Figure 7e, where it can be seen that with 30% MF, there is a 50% reduction in TSR and, with 50% MF, a 70% reduction. This suggests that perhaps some of the aromatic volatiles from degrading UP that burn with a smoky flame are instead incorporated into the char arising from the MF component. It can be seen from the data in Table 6 that yields of residues from the UP/MF blends are greater than expected on the basis of the yields of residue from UP and MF separately.

The flammability of the glass fibre-reinforced composites from these resins was also studied by cone calorimetry; the HRR, mass loss, and RSR vs. time curves of all samples are shown in Figure S3a2–c2, and the derived results are presented in Table 7. In Table 7, the composition of composites is also given, where it can be seen that resin content in all samples is very similar; hence, the results can be compared. The trends for all parameters are very similar to those seen for the cast resins discussed above. On comparing the cone calorimetric results for composites with those for cast resins, it can be seen that there is no significant change in TTI, which is because ignition arises from the resin on the surface, and the surface layers of the composites are rich in resin. The PHRR, THR, and RSR values are much reduced in composites compared with those of the respective resins because of the reduced resin content in the former and thermal barrier effect of the glass fibre. To observe the effect of MF content on cone calorimetric parameters, the % change in each parameter with respect to UP composite vs. MF content is plotted in Figure 7, where it can be seen that, while the absolute values for the composites are slightly different than those for the respective resins, the effect of MF on reduction in flammability is similar in both. With 50% MF in a UP/MF blend, 34% reduction in PHRR, 12% in THR, and 77% reduction in smoke production (TSR) were observed.

### 3.5. Mechanical Properties of Composite Laminates

The mechanical testing of GFRCs of UP, MF, and UP/MF:50/50 blends was performed in tensile, flexural, and impact modes. Since the resin content and, hence, the fibre volume fractions (FVF) of these composites are slightly different, the tensile and flexural results were normalized to 40% FVF. Both original and normalized results are given in Table 8

**Figure 8:**
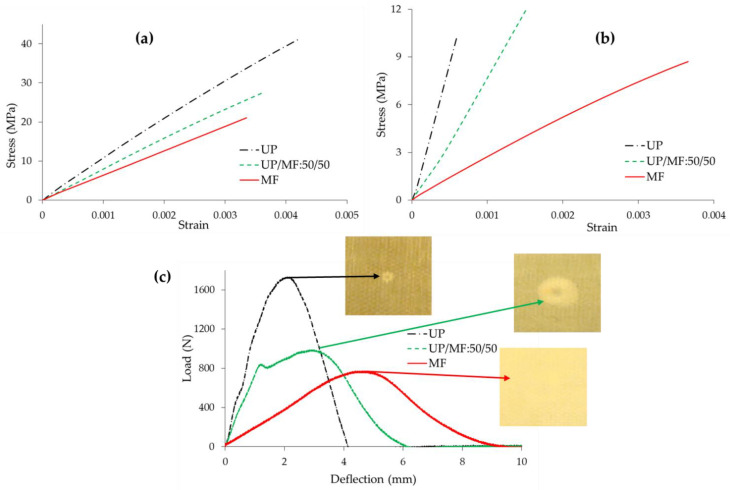
(**a**,**b**) Stress vs. strain curves under (**a**) tensile, (**b**) flexural; and (**c**) load vs. deflection curves under impact load of 1.02 kg and 100 mm drop height, with digital images after the impact test of GFRC samples of UP, UP/MF:50/50, and MF.

The stress vs. strain curves of the GRFCs under tensile load are shown in Figure 8a. The slope of stress–strain curves for UP, MF, and the UP/MF:50/50 GRFCs are very different (see Figure 8a). As can be seen from Table 8, the tensile modulus of MF is much lower than that of the UP. The modulus of the 50/50 blend, 8.4 GPa, is lower than expected from application of the rule of mixtures (9.6 GPa); this may be because of heterogeneity in the resins in the blend or poor adhesion between the matrix and the fibre. MF resins are mainly used in surface coatings, textile finishes, and laminate floorings and have poor tensile properties.

The stress vs. strain curves of the GRFC under three point bending mode in Figure 8b also show poor performance of MF and, hence, the blend. The 3.7 GPa flexural modulus of MF is much lower than that of UP (19 GPa). The flexural modulus is resin-dependent, and because of the low flexural modulus of the MF resin, in the UP/MF:50/50 composite laminate, the flexural modulus value is reduced by >50% compared with that of UP. In addition, MF may have a plasticising effect on the UP because of the DEG solvent in the MF resin. Since it is the matrix that mainly governs the flexural properties of a GRFC, it is suggested that the blended resin matrix does not act as a single material and has poor interfacial adhesion to the glass fibres in the composite laminates. This conjecture is supported by the DMTA and SEM results discussed earlier.

The composite laminates were also tested under dynamic loading using an impact drop weight of 1.02 kg from a height of 100 mm in an Instron Dynatup tester. From the load vs. deflection impact curves of the composite samples in Figure 8c, the impact modulus (toughness), amount of energy absorption, and the damage caused by the impact loading have been calculated. After impact testing, the damaged area on the tested sample was also measured; digital images of the samples are also shown in Figure 8c. From Figure 8c, it can be clearly seen that the load vs. deflection curve of UP is smooth and uniform. The impact modulus of the UP is 19.6 GPa, and the damage caused by the drop weight on the surface of the laminate (inset image in Figure 8c) has a ~3 mm diameter. The broader smooth curve for MF indicates that it has the potential to absorb more energy than UP and is more flexible than UP. The impact modulus of MF is only 2.7 GPa. The damage caused by the drop weight during impact test is not visible in MF, indicating that the resin is more plastic. In Figure 8c, the initial tangent of curve is almost similar for UP and UP/MF:50/50 GFRC laminates. The impact modulus of UP/MF:50/50 is only 10.1 GPa. However, the shape of the UP/MF:50/50 curve is more similar to that of UP than that of the MF composite. The size of the damage in UP/MF:50/50 is ~14 mm diameter, which is mainly due to phase separation in the blend. 

Overall, these results indicate that the inclusion of MF in UP has significantly impaired the mechanical properties in all modes. This is due to the phase separation in UP/MF blends and to the poor mechanical properties of MF resin itself.

## 4. Conclusions

From this work, it can be concluded that the MF resin cannot be homogeneously co-blended with UP resin. SEM results reveal the existence of two different phases in the blended samples. There is no chemical interaction between UP and MF judging from IR spectra of gases evolved during thermal degradation. From the above results, it can be concluded also that there is no strong physical interaction between the UP and MF matrices at normal temperatures. The thermal stabilities of blended resins are greater than that of UP only above 400 °C, and in the gases evolved during the degradation process, there are no new products compared to those from the pure resins, again indicating no chemical interaction between UP and MF in the blend. However, char yields are slightly greater in the blends than would be expected on the basis of their compositions. The LOI and cone calorimetric results have shown that the presence of MF in the UP/MF resin improves the fire retardancy of the blended material. The flammability of the blends decreases with increasing MF content. The most striking effect of the incorporation of MF in a UP resin is on smoke production, which even with 30% MF content in the blend, is reduced by more than 50%, perhaps owing to some of the smoke-producing aromatic degradation products of UP being incorporated into the MF char rather than being released into the gas phase.

Although the UP/MF co-blended resin systems with different UP/MF ratios show improved fire retardancy, the mechanical properties of the composites are worse than those of UP because of the weak interface between the UP and MF phases and because cured MF itself has poor mechanical properties. However, UP/MF blended resins show promise for applications in which mechanical performance is less important but improved flame retardance is required, such as in coatings. UP/MF resin blends are easy to prepare and viscosities of the blends prior to curing are similar to that of UP itself; they are thus easy to apply as FR coatings, for example, as gel coats to composites.

## Figures and Tables

**Table 1:** Sample composition and curing conditions.

Resin	Curing Conditions
UP	RT 24 h, 80 °C 6 h
MF	RT 24 h, 50 °C 24 h, 80 °C 12 h
UP/MF:70/30	RT 24 h, 80 °C 24 h
UP/MF:50/50	RT 24 h, 80 °C 24 h

**Table 2:** DSC peak details and heat flow for uncured UP, UP/MF blends, and MF resin.

Uncured Sample	Exothermic Peak (C, Figure 1) (°C)		Endothermic (°C)				Heat of Reaction (Exotherm) (J/g)
			Peak1 (A, Figure 1)		Peak 2 (B, Figure 1)		
	T_onset_	T_peak_	T_onset_	T_peak_	T_onset_	T_peak_	
UP	30	82	-	-		-	282
SD-MF	-	-	30	75	132	166	-
MF *	-	-	30	134	159	185	-
UP/MF:70/30	30	74	-	-	137	167	197
UP/MF:50/50	30	80	-	-	134	207	88

Note: * MF = SD-MF dissolved in DEG.

**Table 3:** *T_g_*s and storage moduli of cured cast resin samples of UP and UP/MF blends from DMTA.

Sample	*T_g_* (°C)	Storage Modulus at 40 °C (MPa)
UP	94	2122
UP/MF:70/30	80	913
UP/MF:50/50	77	1044

**Table 4:** TGA, DTG, and DTA analyses of cured UP and MF resins, and their blends in air and nitrogen.

Sample	Air						Nitrogen				
	Temp Range (°C)	Mass Loss (%)	DTG Max (°C)	DTA Max (°C) (Exo)	CY (%)		Temp Range (°C)	Mass Loss (%)	DTG Max (°C)	DTA Max (°C) (Exo)	CY (%)
UP	RT–183	0.9					RT–183	0.9			
	183–435	93.1	373	352(En.S):404	0.4		183–462	94.8	383	369	4.3
	435–566	5.6	532	533							
MF	RT–110	6.3					RT–110	2.3			
	110–288	39.4	173,218	218 (En.S)			110–287	31.7	246	250	
	288–382	25.7	356	378 (En.S)	13.1		287–383	36	358	367	21.5
	382–639	26.0	597	593			383–524	9	472	474	
UP/MF:70/30	RT–206	5.1					RT–276	14.5	220		
	206–437	78.4	363	384 (En.S)	4.4		276–463	74.9	366	377	9.0
	437–605	15.6	559	556							
UP/MF:50/50	RT–206	9.9					RT–276	22	221		
	206–475	73.9	363	376 (En)	7.4		276–453	63	373	383	10.9
	475–627	16	576,621	573,623							

Note: En. Indicates endothermic, S indicates small and Exo indicates exothermic. CY = Residue at 575 °C.

**Table 5:** Quantities of evolved gases calculated from TGA-FTIR spectra of UP, MF, and their blends in nitrogen.

Sample	Gas Evolved (FTIR Peak((cm^−1^) × 10^−2^))							
	H_2_O	CH_3_OH	Ali.Hyd. Carbons	CO_2_	Phthalic Anhydride	Styrene	NH_3_	HNCO
	(3750)	(1035)	(2925)	(2360)	(1866)	(700)	(965)	(2284)
UP	0.3	0.89	1.49	1.91	2.68	2.53	0	0
UP/MF:70/30	0.64 *0.71*	2.74 *1.78*	1.94 *1.90*	1.33 *1.90*	1.43 *1.88*	1.12 *1.77*	1.69 *1.53*	0.94 *0.89*
UP/MF:50/50	1.1 *0.98*	3.04 *2.38*	2.3 *2.18*	1.03 *1.89*	0.72 *1.34*	1.00 *1.27*	2.72 *2.56*	1.58 *1.49*
MF	1.65	3.86	2.86	1.87	0	0.00	5.11	2.97

Note: The values in red and in italics are the calculated expected sums of those from the individual components.

**Table 6:** Flammability results for cast resin samples of UP, MF, and UP/MF blends.

Sample	LOI (%)	Cone Calorimetric Results at at 50 kW/m^2^ Heat Flux					
		TTI (s)	FO (s)	PHRR (kW/m²)	THR (MJ/m^2^)	TSR (m²/m²)	Residue (%)
UP	17.9 ± 0.1	36	188	1110	83	4813	1.3
UP/MF:70/30	18.4 ± 0.2 *(19.2)*	30	192	822	73	2385	8.4
UP/MF:50/50	19.2 ± 0.1 *(20.0)*	22	196	674	71	1832	11.1
MF	22.1 ± 0.2	20	198	513	48	0	18.9

Note: The variation in values for different parameters are: TTI (time-to-ignition) = ±2; FO (flame out) = ±5; PHRR (peak heat release rate) = ±37; THR (total heat release) = ±1.6; TSR (total smoke released) = ±126; CY (char yield) % = ±3.2. The LOI values in red and parentheses are calculated from those of the components

**Table 7:** Composition and flammability results of GFRC composites of UP, MF, and UP/MF: 50/50 blend.

Sample	Mass Fraction (%)			Cone Calorimetric Results at 50 kW/m^2^ Heat Flux					
	Glass	Resin		TTI (s)	FO (s)	PHRR (kW/m²)	THR (MJ/m^2^)	TSR (m²/ m²)	Residue (%)
UP	57	43		34	128	599	33	1640	57
UP/MF:50/50	58	42		22	127	395	29	380	60
MF	55	45		14	121	361	26	0	65

Note: The variation in values for different parameters are as TTI (time-to-ignition) = ±2; FO (flame out) = ±3; PHRR (peak heat release rate) = ±24; THR (total heat release) = ±0.5; TSR (total smoke release) = ±41; residue = ±0.8.

**Table 8:** Mechanical properties of GFRCs of UP, MF, and their 50/50 blend.

Sample	Thick. (mm)	FVF	Tensile Modulus (GPa)			Flexural Modulus (GPa)			Impact Modulus (GPa)
	
			Original	Normalised *		Original	Normalised *		
UP	2.4	35.2	11.1 ± 0.6	12.6		15.8 ± 0.6	18		19.6 ± 0.0
UP/MF:50/50	2.5	39.0	8.2 ± 0.2	8.4		8.5 ± 0.4	8.7		10.1 ± 0.1
MF	2.5	38.2	6.4 ± 0.3	6.7		3.5 ± 0.2	3.7		2.7 ± 0.1

Note: FVF = fibre volume fraction. * = Normalised with respect to 40%FVF

## Data Availability

The data presented in this study are available from the corresponding author upon request.

## Supplementary Materials

The following supporting information can be downloaded at https://www.mdpi.com/article/10.3390/polym14224885/s1, Figure S1: (a1,a2) TGA curves and (b1,b2) Derivative weight loss curves and (c1,c2) DTA curves of cured UP, MF resin and their blends; (a1–c1) in air and (a2–c2) in nitrogen; Figure S2: FTIR spectra of evolved gases in nitrogen: (a) UP, (b) MF, and (c) UP/MF:50/50 at different temperatures; Figure S3: Plots of (a1,a2) HRR; (b1,b2) mass loss and (c1,c2) RSR vs. time for UP, MF, and UP/MF:50/50 (a1–c1) cast resins and (a2–c2) GFRC samples at 50 kW/m^2^ external heat flux.
