# Supplementary material for: Thermal Stability, Flammability and Mechanical Performances of Unsaturated Polyester–Melamine Resin Blends and of Glass Fibre-Reinforced Composites Based on Them
Source: Polymers (Basel). 2022 Nov 12;14(22):4885. doi: 10.3390/polym14224885 (PMC9693295; doi:10.3390/polym14224885)
Supplement: Supplementary file 1 [file polymers-14-04885-s001.zip › polymers-1974874-supplementary.pdf]

polymers

Article

Thermal stability, flammability and mechanical performances of unsaturated polyester-melamine resin blends and of glass fibre-reinforced composites based on them

Latha Krishnan[1], Baljinder K Kandola[1]*, Dario Deli1[1,2] and John R Ebdon[1]

[1] Institute for Materials Research and Innovation, University of Bolton, Deane Road, Bolton, BL3 5AB, UK
[2] Present address: Romer Labs UK Ltd, The Heath Business & Technical Park, Runcorn, WA7 4QX, UK
* Correspondence: B.Kandola@bolton.ac.uk; Tel.: +44-1204903517

Supplementary data

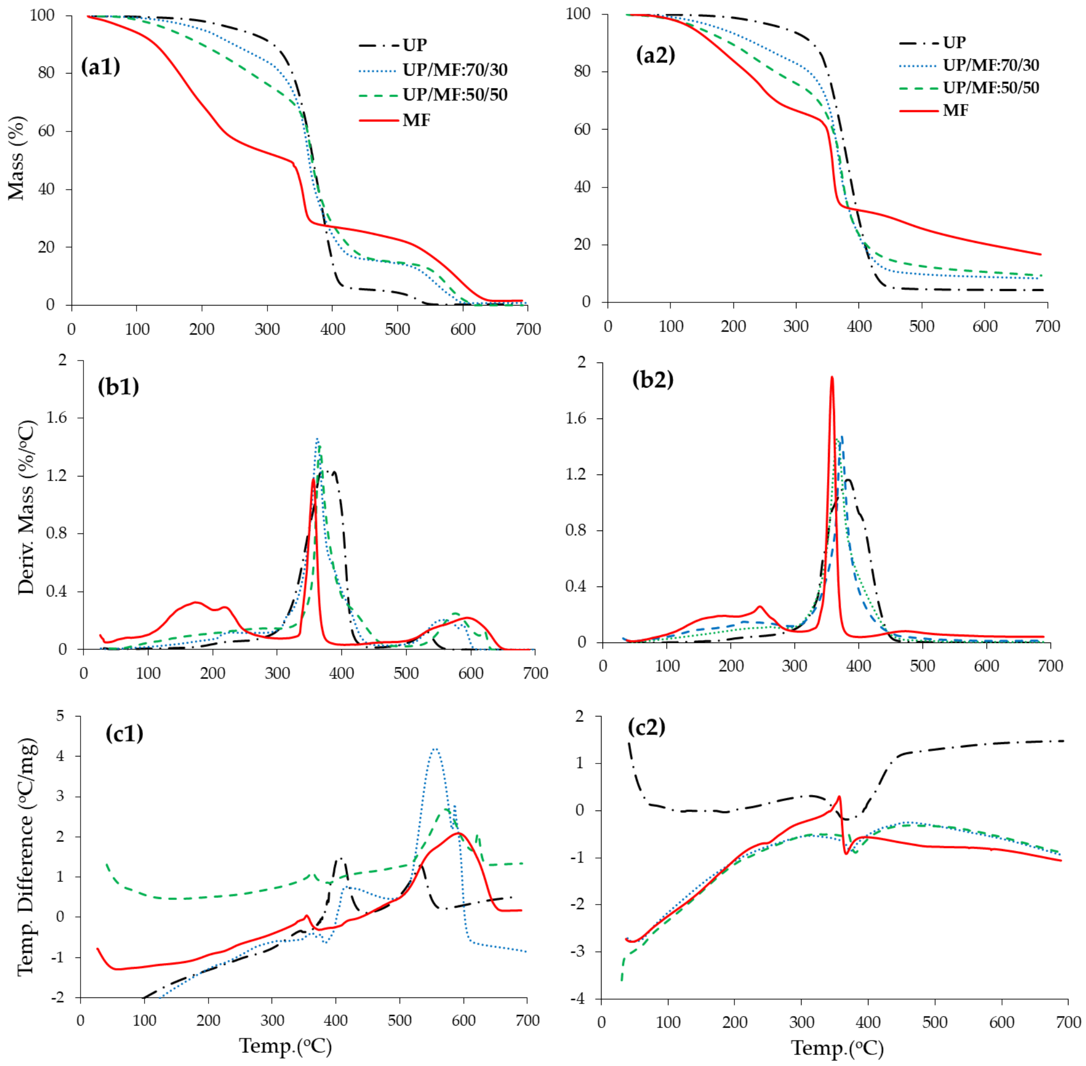

Figure S1. (**a1**), (**a2**) TGA curves and (**b1**), (**b2**) Derivative weight loss curves and (**c1**), (**c2**) DTA curves of cured UP, MF resin and their blends; (**a1**–**c1**)) in air and (**a2**–**c2**)) in nitrogen

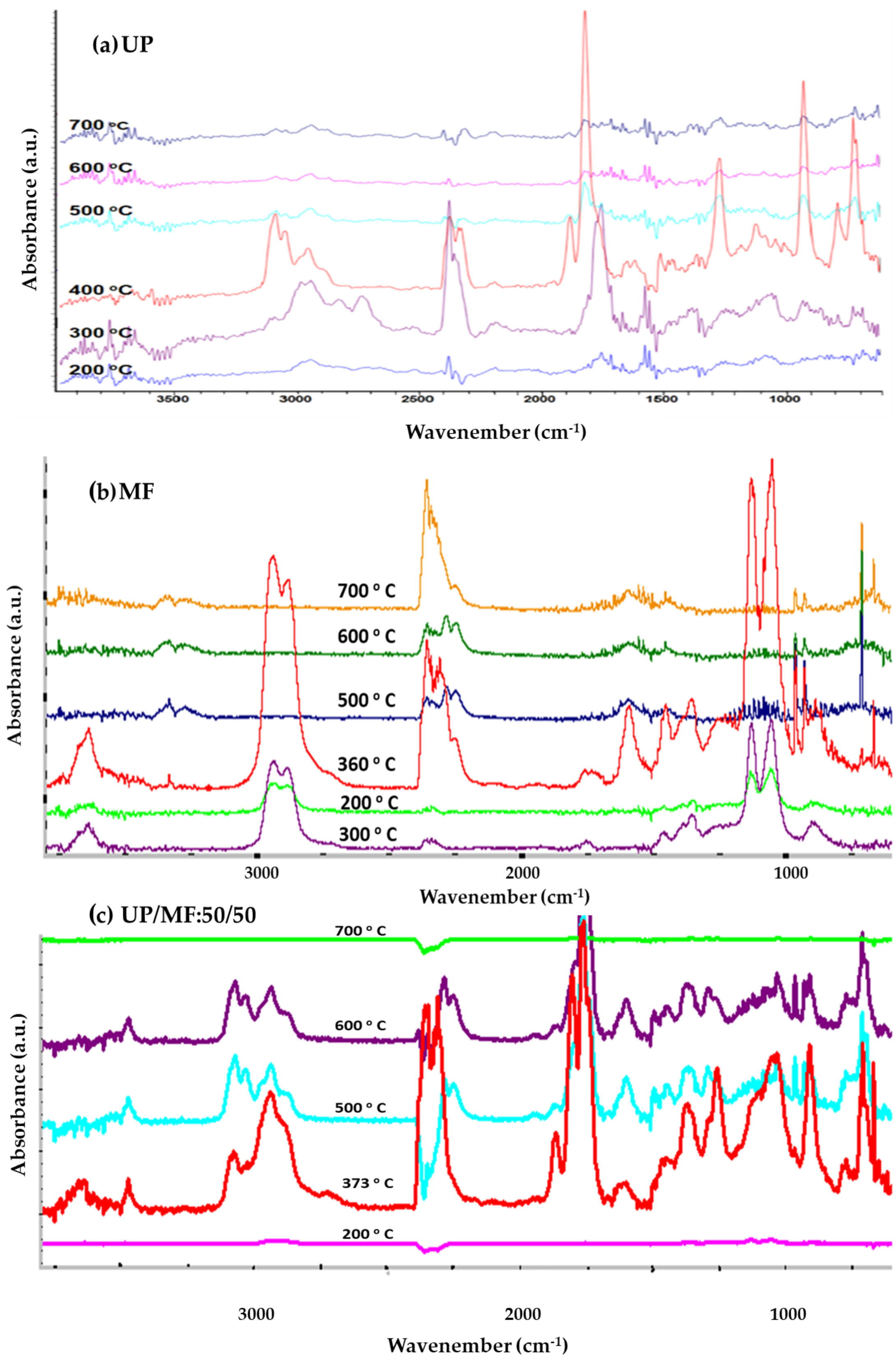

Figure S2. FTIR spectra of evolved gases in nitrogen: (**a**) UP, (**b**) MF and (**c**) UP/MF:50/50 at different temperatures

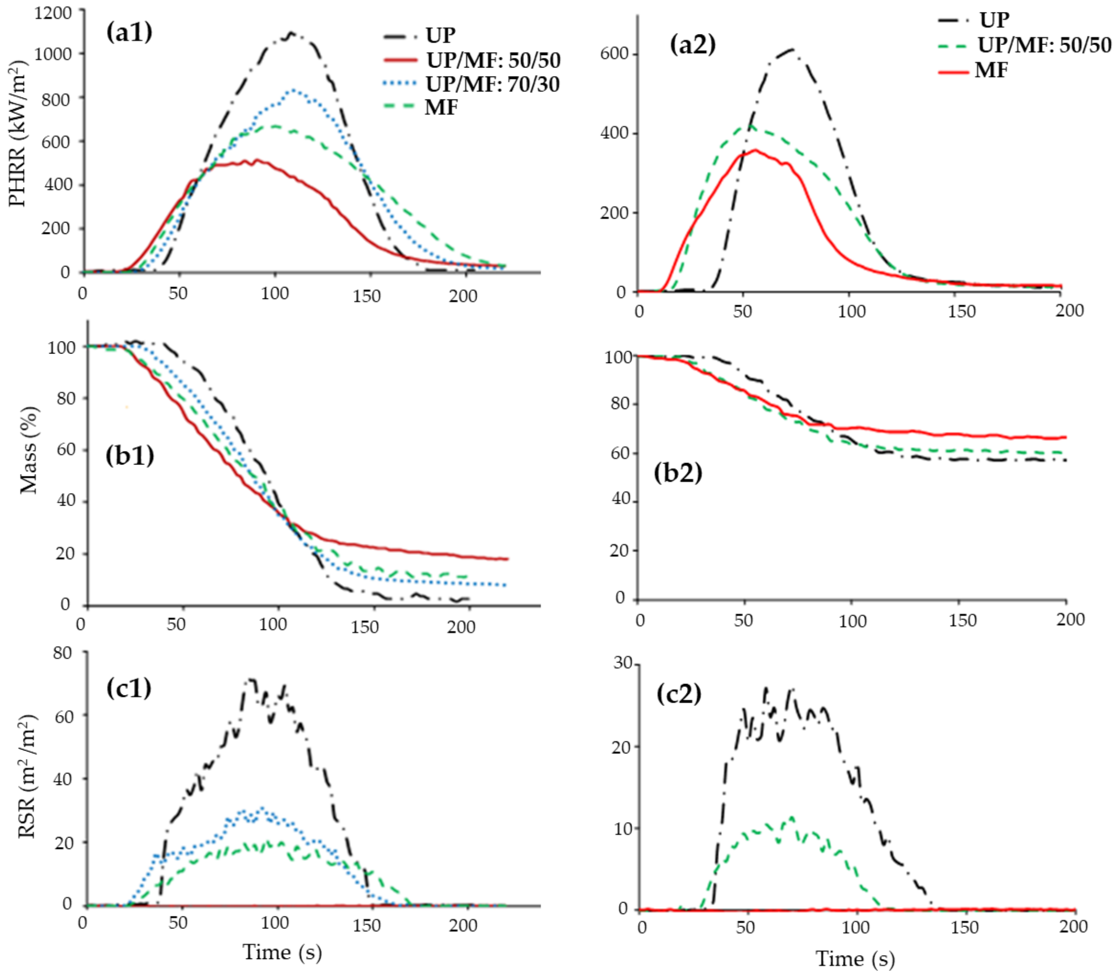

Figure S3. Plots of (**a1**,**a2**) HRR; (**b1**, **b2**) mass loss and (**c1**,**c2**) RSR *vs.* time for UP, MF and UP/MF:50/50 (**a1**–**c1**) cast reains and (**a2**–**c2**) GFRC samples at 50 kW/m² external heat flux
